# Testosterone Therapy in Men with Klinefelter Syndrome: Analysis of a Global Federated Research Network

**DOI:** 10.1089/andro.2022.0020

**Published:** 2022-12-28

**Authors:** Chase Carto, Justin Loloi, Katherine Campbell, Ranjith Ramasamy

**Affiliations:** ^1^ Desai Sethi Urology Institute, Miller School of Medicine, University of Miami, Miami, Florida, USA.; ^2^ Department of Urology, Montefiore Medical Center, Albert Einstein College of Medicine, Bronx, New York, USA.

**Keywords:** Klinefelter, testosterone, therapy, prescription, hypogonadism

## Abstract

**Introduction:**

The objective of this study was to determine the rates of hypogonadism and prescription of testosterone replacement therapy (TRT) in men with Klinefelter syndrome (KS). We hypothesized that men with KS are under-treated for testosterone deficiency with TRT due to a combination of factors, including a poor understanding of hypogonadism in this population and neurocognitive issues leading to delay in seeking of treatment for hypogonadism.

**Materials & Methods:**

We queried TriNetX, a large multicenter electronic health record database, to identify all men with a diagnosis of KS (ICD-10-CM Q98.4). Prevalence of testosterone deficiency was determined as defined by testosterone level < 300 ng/dL. The primary outcome of the study was prescription of any of the following forms of TRT on the day of diagnosis or later.

**Results:**

There were in total 5437 men with diagnosis of KS. A total of 1581 men with KS received laboratory measurement of testosterone level, 1113 (70.4%) of whom were hypogonadal. Mean testosterone level in this group was 354 ng/dL [50–658]. Of the 1113 men found to be hypogonadal, only 657 (59.0%) men were given prescription for TRT.

**Discussion & Conclusion:**

This is the first study to evaluate TRT prescribing habits in men with KS. In this large retrospective study, TRT was underprescribed in men with KS. Further studies are needed to corroborate these findings and to evaluate barriers to receiving care in this population.

## Introduction

Klinefelter syndrome (KS, 47 XXY) represents the most common genetic form of male hypogonadism.^1^ It has an estimated prevalence of 1.72 per 1000 male births, with that number thought to be increasing.^2^ The key findings in KS include small testes, cognitive impairment in the area of language, and hypergonadotropic hypogonadism.^3^ Despite being well described, the clinical phenotype is highly variable and it is rare to observe all the attributes of this characteristic phenotype in the clinical setting.^4^ Therefore, as many as 50–75% of men with KS may be inaccurately diagnosed or undiagnosed altogether leaving them vulnerable to medical, psychological, and social issues.^5^

Most patients with KS have normal prepubertal development but develop fibrosis of the interstitium and Leydig cell hyperplasia leading to the diminished testis size and subsequent hypogonadism characteristic of KS.^6^ Of note, hypogonadism is often not present at the time of diagnosis and may develop over time depending on level of circulating testosterone.^7^ Given the degree and prevalence of hypogonadism in these men, it is recommended that KS patients with documented hypogonadism should be treated with lifelong testosterone supplementation that begins at puberty.^8^ Testosterone replacement therapy (TRT) in men with KS should secure development of sexual male characteristics and prevent long-term sequelae of chronic hypogonadism.^9^

Although hypogonadism is among the classic characteristics of KS, the effects of TRT are not well studied, and despite the recommendation for TRT use in KS patients with hypogonadism, there is a scarcity of data describing whether this is routinely employed in clinical practice. In this study, we aimed to investigate the prevalence of hypogonadism in men with KS, and to describe the rates of TRT prescription among men with KS. We hypothesized that patients with diagnosed KS would be undertreated with TRT.

## Materials and Methods

**FIG. 1.**
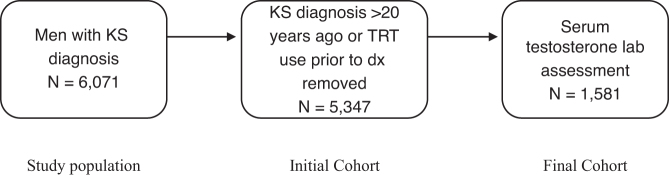
Cohort selection.

We queried TriNetX, a large multicenter electronic health record database, to identify men with a diagnosis of KS (ICD-10-CM Q98.4). Exclusion criteria were then applied to create the initial cohort, which included men with documented use of testosterone before diagnosis of KS or men with diagnosis of KS >20 years ago due to incomplete information available in the electronic health record. Data on comorbidities, including mental retardation (F70-F79), type 2 diabetes mellitus (E11), obesity (E66), hyperlipidemia (E78.5), hypertension (I10), osteoporosis (M81), pulmonary embolism (I26), and other vascular thrombosis (I74), were collected for all patients in addition to demographic characteristics. We then identified a laboratory cohort by identifying men in the initial cohort who had received a testosterone laboratory measurement on the day of diagnosis of KS or later. A brief flowchart detailing the creation of each cohort is shown in Figure 1.

The primary outcome of the study was prescription of any of the following forms of TRT on the day of diagnosis or later using associated medication codes: testosterone, testosterone 17-phenylpropionate, testosterone enanthate, testosterone cypionate, and testosterone undecanoate. In addition, we investigated the prevalence of hypogonadism in men with diagnosis of KS and documented testosterone laboratory measurement, as defined by testosterone level <300 ng/dL.^10^ Rates of TRT prescription among hypogonadal men with KS were assessed separately. IRB waiver was obtained from the University of Miami.

## Results

There were in total 6071 men in the initial cohort with diagnosis of KS in the TriNetX database. After excluding patients with a diagnosis of KS >20 years ago or use of any form of TRT before diagnosis with KS, 5347 patients remained. The majority of men were White (71%), followed by men of unknown race (17%) and African American (9%). Mean age at diagnosis was 33 years [13–53]. Descriptive analysis of comorbidities revealed 350 (6.5%) patients with a diagnosis of intellectual disability, 761 (14.2%) with type 2 diabetes mellitus, 995 (18.6%) with obesity, 919 (17.2%) with hypertension, 246 (4.6%) with osteoporosis, 757 (14.2%) with hyperlipidemia, 153 (2.9%) with history of pulmonary embolism, and 60 (1.1%) with a history of vascular thrombosis.

**FIG. 2.**
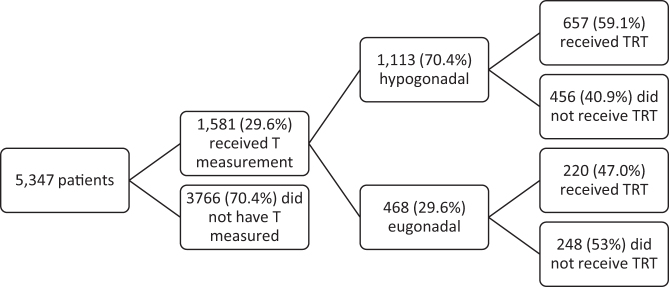
Patients who received T measurement and subsequent treatment.

In total, of these 5347 patients, 1484 (27.8%) were prescribed any form of TRT (Table 1). A total of 1581 men (29.6%) received a laboratory measurement of their testosterone level on the day of diagnosis or later (Fig. 2). Mean testosterone level in this group was 354 [50–658], and 1113 (70.4%) were hypogonadal, as defined by a testosterone level <300 ng/dL. In 1113 men found to be hypogonadal on testosterone laboratory measurement, 456 (41.0%) men did not receive any form of TRT. An additional 220 men (47%) who were found to be eugonadal received TRT.

**Table 1.** Descriptive Analysis of Age, Testosterone Level, and Comorbidity Presence

Characteristics	Total (*N* = 5347)
Age (*μ*) (years)	33 ± 20
Race, *n* (%)	
White	3796 (71)
Black or African American	481 (9)
Asian	107 (2)
American Indian or Alaskan Native	54 (1)
Unknown	909 (17)
Comorbidities, *n* (%)	
Mental retardation	350 (6.5)
Type 2 diabetes mellitus	761 (14.2)
Obesity	995 (18.6)
Hypertension	919 (17.2)
Osteoporosis	246 (4.6)
Hyperlipidemia	757 (14.2)
Pulmonary embolism	153 (2.9)
Other vascular thrombosis, *n* (%)	60 (1.1)
Prescription of TRT	1484 (27.8)
	Subtotal (*N* = 1581)
Total testosterone (μ) (ng/dL)	354 ± 304

TRT, testosterone replacement therapy.

## Discussion

Hypogonadism poses a significant burden on the quality of life of men. Despite the recommendation to provide TRT to hypogonadal men with KS, there is a paucity of data describing real-world prescription of TRT in these men. We aimed to determine treatment rates with TRT in men diagnosed with KS, in addition to the prevalence of hypogonadism in this patient population using a large multi-institutional electronic health record database. We hypothesized that despite recent recommendations for lifelong treatment^11^ in those who are hypogonadal and FDA approval for management of hypogonadism in this population,^12^ men with KS would be undertreated with TRT.

Our study of a large global federated research network highlights that just 27.8% of men with diagnosis of KS subsequently received any form of TRT, and only 26% of men were evaluated further with laboratory measurement of serum testosterone level. We found that only 59.0% of men who were hypogonadal (*T* < 300 ng/dL) by testosterone laboratory assessment received testosterone replacement. Thus, there is significant room for improvement in properly treating men with KS and hypogonadism. To our knowledge, there are no previous studies in the literature that characterize the rates of prescription of TRT for patients with KS. By understanding treatment rates in men with KS, we can begin to explore the social, mental, and clinical considerations in managing these patients.

Smyth and Brenner previously reported that between 65% and 85% of men with KS have decreased testosterone levels, but that 90–100% of men with KS have elevated gonadotropin levels.^13^ In our study, only 26% of men with a diagnosis of KS had a testosterone level measured, but 70.4% of those men were found to be hypogonadal, which is consistent with the previously reported data. Although methods of testosterone measurement and the threshold for considering a man hypogonadal have changed in the 35 years since publication of their study, the prevalence of hypogonadism in men with KS has remained high.^3^

Individuals with KS also experience pervasive neurocognitive deficits, which pose additional challenges for management of their chronic conditions. Patients with KS may experience various degrees impairment in language and executive function (concept formation, problem-solving, and task switching).^14^ An impaired self-awareness and learning difficulties exhibited in some KS patients could potentially lead to limited recognition of the signs and symptoms of hypogonadism, although it remains to be seen if this reliably translates to a lack of capacity to recognize symptoms of hypogonadism.^15^ Without strong family or social support, patients with KS may be less able to seek evaluation or treatment for their condition. This consideration may partially explain the undertreatment of KS patients with TRT.

To our knowledge, this study is the first to describe the rates of TRT prescription in men with KS, and is one of few studies in the literature to objectively describe the prevalence of hypogonadism in patients with KS in a large patient population. Additional strengths of our findings include the large sample size and the ability to establish the chronology of diagnosis and further evaluation and management. However, our findings are not without limitations. First, the retrospective nature of our study may introduce incomplete or inaccurate capture of data. It is also possible that men with a diagnosis of KS followed up for clinical workup and subsequent TRT prescription at a health care organization that does not report data to the TriNetX database, potentially leading to underestimation of the true rate of treatment with TRT in this cohort.

Although we assume that physicians only provided TRT to men who were found to be hypogonadal, this database does not allow us to draw the conclusion that all men treated with TRT were hypogonadal. In addition, there are no data available on if patients experienced symptoms associated with hypogonadism in our cohort. It is possible that men who were asymptomatic were less likely to receive treatment with TRT even if they were hypogonadal by laboratory assessment. Furthermore, this study lacks data on clinical follow-up, so we are unable to assess whether or not these men were undertreated due to the fact that they did not follow up with a specialist to discuss their diagnosis or laboratory results and receive TRT.

## Conclusion

In this retrospective epidemiological study, we found that men with a diagnosis of KS are undertreated with TRT despite the recommendation for lifelong testosterone replacement after puberty in this population. In a large cohort of 5347 patients with KS, only 27.8% subsequently received TRT. Furthermore, a majority of hypogonadal KS patients were not treated with TRT. Future studies are needed to corroborate our findings. We are especially interested in further study into other problems that men with KS face such as sexual dysfunction. In particular, research exploring the psychological, social, and socioeconomic considerations in management of patients with KS could shed light on important underlying mechanisms for underdiagnosis and undertreatment in this population.

## Abbreviations Used

KS: Klinefelter syndrome
TRT: testosterone replacement therapy
